# Delayed Graft Function in Kidney Transplants: Time Evolution, Role of Acute Rejection, Risk Factors, and Impact on Patient and Graft Outcome

**DOI:** 10.1155/2015/163757

**Published:** 2015-09-10

**Authors:** Martin Chaumont, Judith Racapé, Nilufer Broeders, Fadoua El Mountahi, Annick Massart, Thomas Baudoux, Jean-Michel Hougardy, Dimitri Mikhalsky, Anwar Hamade, Alain Le Moine, Daniel Abramowicz, Pierre Vereerstraeten

**Affiliations:** ^1^Department of Nephrology, Dialysis and Transplantation and Department of Abdominal Surgery, CUB, Erasmus Hospital, Route de Lennik 808, 1070 Brussels, Belgium; ^2^Research Center of Biostatistics, Epidemiology and Clinical Research, School of Public Health, Route de Lennik 808, 1070 Brussels, Belgium; ^3^Nephrology Department, Antwerp University Hospital, Free University of Brussels, Wilrijkstraat 10, 2650 Edegem, Belgium

## Abstract

*Background*. Although numerous risk factors for delayed graft function (DGF) have been identified, the role of ischemia-reperfusion injury and acute rejection episodes (ARE) occurring during the DGF period is ill-defined and DGF impact on patient and graft outcome remains controversial. *Methods*. From 1983 to 2014, 1784 kidney-only transplantations from deceased donors were studied. Classical risk factors for DGF along with two novel ones, recipient's perioperative saline loading and residual diuresis, were analyzed by logistic regression and receiver operating characteristic (ROC) curves. *Results*. Along with other risk factors, absence of perioperative saline loading increases acute rejection incidence (OR = 1.9 [1.2–2.9]). Moreover, we observed two novel risk factors for DGF: patient's residual diuresis ≤500 mL/d (OR = 2.3 [1.6–3.5]) and absence of perioperative saline loading (OR = 3.3 [2.0–5.4]). Area under the curve of the ROC curve (0.77 [0.74–0.81]) shows an excellent discriminant power of our model, irrespective of rejection. DGF does not influence patient survival (*P* = 0.54). However, graft survival is decreased only when rejection was associated with DGF (*P* < 0.001).  *Conclusions*. Perioperative saline loading efficiently prevents ischemia-reperfusion injury, which is the predominant factor inducing DGF. DGF *per se* has no influence on patient and graft outcome. Its incidence is currently close to 5% in our centre.

## 1. Introduction

Delayed graft function (DGF) is a common complication most frequently defined as the need for dialysis during the first posttransplant week [1]. DGF results from immunologic and nonimmunologic events that start during kidney preservation and progress after the time of reperfusion [2]. DGF incidence depends on its definition [1], on the risk profiles of the donor and the recipient [3], and on the transplant centre [4]. Over the past decade, there have been important advances in the field of transplantation particularly with regard to immunosuppression [5] and organ allocation strategies [6] leading to reduced DGF incidence but it is expected to rise in the future due to the increasing use of kidneys from extended criteria and donation after cardiac death (DCD) donors who show more DGF versus standard criteria donors [7]. In the last thirty years, several studies report DGF incidences in adult recipients from deceased kidney donor transplants to be within the 15–30% range [3].

In 2003, Irish et al. developed the first multivariable logistic regression model to quantify the risk of DGF using donor and recipient characteristics at the time of transplantation [3] and in 2010 they refined their model and found that the main risk factors for DGF are cold ischemia time, donor's terminal serum creatinine, recipient body mass index (BMI), DCD donor, and donor's age ≥16 years [8]. In recent years, although much effort has been made to identify the risk factors for DGF, its cause was scarcely investigated. Ischemia-reperfusion injury and acute rejection episodes occurring during the DGF period are both the main causal mechanisms of DGF and they are hard to distinguish from each other as they are highly correlated [9]. Furthermore, the impact of DGF on patient and graft outcome is controversial due to lack of control for acute rejection in most analyses. Some studies found shorter allograft survival regardless of rejection occurrence [10–12] but others did not [13, 14]. Altogether, DGF seems to decrease long-term graft survival as a result of combined ischemia-reperfusion injury and early rejection [15].

Given the burden of DGF impact, many strategies are now at different stages of development, which might reduce the risk of DGF in the future, but there are no approved strategies for DGF prevention to date [7]. Of course, some factors could be modified: cold ischemia time should be kept as low as possible [14] and immunosuppressive therapy adapted to the patient with high DGF risk [14, 16], but these modifications are not always feasible. Readily accessible methods to minimize ischemia-reperfusion injury include, for example, maintenance of an adequate patient's perioperative intravascular volume [17]. Surprisingly, predictive models of DGF do not take the recipient's perioperative hemodynamics into account [3, 8, 18, 19].

In our centre, DGF incidence dropped steeply in 1992 and subsequently decreased continuously until reaching a record low since 2010, close to 5%. Our aim was to answer four questions: (1) What are the factors behind the decrease in DGF incidence in our cohort across the three past decades? (2) What role does rejection play in DGF occurrence? (3) What are the risk factors for DGF taking rejection into account or not? (4) Does DGF impact patient and graft outcome differently with and without rejection?

## 2. Patients and Methods

From August 25, 1983, when cyclosporin was first used at our centre, to June 30, 2014, which defines the end of the study period, 1784 consecutive kidney-only transplantations from deceased donors have been carried out on 1452 adult (≥15 years old) recipients. The immunosuppressive protocol on an intent-to-treat basis included, along with corticosteroids, a calcineurin inhibitor either cyclosporin (*n* = 1046) or tacrolimus (*n* = 690) and an antiproliferative medication either azathioprine (*n* = 191) or mycophenolate mofetil (*n* = 684) and it was associated in most cases with an antilymphocyte induction therapy consisting in either a polyclonal globulin (antithymocyte, *n* = 285) or a monoclonal globulin directed to the CD3 receptor (OKT3, *n* = 578) or to the IL-2 receptor (basiliximab or daclizumab, *n* = 605). A few patients received sirolimus (*n* = 48) and others an anti-LFA1 induction therapy (*n* = 13). Few transplantations (*n* = 32) were preemptive.

Since 1992, a protocol of perioperative saline loading of the recipient has been implemented in our center to prevent hypovolemia [20]. First, during a period of 3 to 4 hours prior to transplantation, the patient receives infusion of 0.9% NaCl; the volume is adapted to the state of hydration and, in case of euvolemia, maintained at a 30 mL/h rate. Second, 0.9% NaCl is infused in the operating room at a maximal 600 mL/h rate until target central venous pressure between 4 and 8 mmHg is obtained and then reduced to 30 mL/h to maintain this pressure. During the first eight hours after transplantation, if recipient's diuresis is more than 20 mL/h, it is compensated by 0.45% NaCl-5% glucose infusion at a maximal 200 mL/h rate, and if diuresis is lower than 20 mL/h, furosemide (250 mg IV) is given and the infusion reduced to a 30 mL/h rate. After the first eight hours, 500 or 1000 mL of the same infusion is given every 12 hours if diuresis is less or more than 20 mL/h, respectively. All preservation infusions used for the cold storage were of the intracellular type and penicillin, insulin, and hydrocortisone were at times added.

Rejection episodes occurring during the DGF period were recorded. The diagnosis was established, first from clinical data (decrease in diuresis, swelling and tenderness of the graft, and malaise) and then from the rise in serum creatinine [21], and finally confirmed by kidney biopsy which has been almost systematic since the early nineties and characterized according to the Banff classification since 1998. These rejection episodes (including Banff borderline ARE) were all treated with transitorily increased corticosteroid doses or, in a few cases of corticoresistance, with thymoglobulin and/or plasmapheresis or, more recently, with intravenous immunoglobulin administration when CD4 positive cells expressing humoral rejection were identified.

DGF was defined as the need for dialysis in the postoperative course irrespective of its duration. Grafts that never functioned due to technical failures, arterial and/or venous thrombosis (*n* = 33), primary nonfunction (*n* = 9), or urological problems (*n* = 8), were not considered as DGF, as regular dialysis was not interrupted.

Most recipient, donor, and transplant characteristics reported in the literature as risk factors for DGF were considered [3]. Some of them were rather infrequent in our centre and were therefore not taken into account, such as recipient's ethnicity (3% black Africans), machine-perfusion preservation (0.3%), and extended criteria donors (6.3% in 2013) [22], and were therefore not considered in further analyses. Besides a recipient's hypertension history which is often ill-defined, all recipient's cardiovascular antecedents (hypertension, coronary heart disease, proximal or distal arteritis of lower limbs, transient ischemic cerebral episodes, or strokes) were collected as a risk factor for DGF. Besides donor's weight, the more accurate BMI was computed. Moreover, recipient's residual diuresis at the time of transplantation was also considered as a potential risk factor for DGF; indeed, if the function of native kidneys is not negligible but requires regular dialyses, one would expect the need of posttransplant dialysis to be lower.

Causes of donor's death were distributed into two main groups: traumatic, resulting from traffic accident, fall, or gunshot, and nontraumatic, due to stroke, anoxia, or brain tumor.

Several demographic characteristics commonly considered as potentially influencing patient and graft survival were recorded: gender and age of the recipient and the donor, number of the graft and pretransplant blood transfusions, duration of the pretransplant regular dialysis period, peak anti-HLA sensitization, cold and warm ischaemia times, DGF, number of HLA mismatches, oral immunosuppression, and induction therapy with poly- or monoclonal globulins.

All the data were collected from our own database or from that kindly provided by the Eurotransplant International Foundation which organizes all kidney exchanges within eight west-European countries including ours [22].

*Statistical Methods*. Fisher's exact test or chi-square tests were used to compare groups with nominal variables and *F* tests groups with numerical variables. Results were expressed as percent of the total for nominal variables and as mean and standard deviation for numerical variables.

A multivariate binary logistic regression model was used to identify the risk factors for rejection and DGF. A backward elimination procedure, based on likelihood ratio, was used to select variables to include in logistic regression models. The Hosmer-Lemeshow test was used to estimate the goodness of fit of our model. Continuous variables were categorized to test the linearity of the log odds scale computed from the logistic regression equation, and if nonlinear trends were identified for some variables, instead of introducing a quadratic term, a dichotomous category with special interest was created. Adjusted ORs and 95% CIs were derived from the final logistic models.

A receiver operating characteristic (ROC) curve was built from the results of the logistic regression in order to estimate the discriminant power of the factors which emerged from the logistic regression model. An area under the ROC curve and its 95% confidence interval were computed.

Univariate survival was computed according to the Kaplan-Meier method and the Cox proportional hazard method was used in multivariate survival analyses.

StatView [23] and Stata [2] statistical software packages were used in all analyses.

The study has been reviewed by the local ethics committee and has therefore been performed in accordance with the ethical standards of the Declaration of Helsinki 2000 as well as the Declaration of Istanbul 2008.

## 3. Results

### 3.1. Time Evolution of Risk Factors for DGF

The incidence of nearly all risk factors changed significantly in the course of time (Table 1). These changes occurred sometimes at random such as for regrafting and duration of the pretransplant dialysis period; some supervened abruptly such as for immunosuppression and perioperative saline loading, but most other changes were gradual, departing or not from linearity. Many risk factors were highly (*P* < 0.001) correlated: for instance, regrafting with peak HLA sensitization, immunosuppression and HLA mismatches with acute rejection episodes, and DGF with cold ischemia time. Grafts with missing data were predominantly encountered in the 1983–1997 period.

### 3.2. Incidence of DGF

Altogether, DGF was observed in 382 out of 1784 transplantations (21.4%) and its duration was limited to the first two posttransplant weeks in 81.4% of the cases (Table 2). Transplants in which acute rejection occurred during the DGF period had a significantly longer duration of DGF than those free of rejection (*P* < 0.001), the median difference between both groups being 5.5 days.

### 3.3. Causes of Donor's Death

The cause of death was unknown in 100 transplants (5.6%). Five groups of known causes were considered: traumatism resulting from traffic accident, fall, or gunshot, cerebrovascular event (haemorrhagic or thrombotic), anoxia due to respiratory arrest induced by drugs or severe asthma, brain tumor, and other less frequent miscellaneous causes. By far, the first two causes were predominant (90.8%) and a clear relationship appeared between the cause of death and donor terminal creatinine, distinguishing traumatic from nontraumatic causes (Table 3): donor's terminal creatinine was significantly higher in donors dead from trauma (*P* < 0.001) than in those dead from other causes (*P* = 0.74).

### 3.4. Risk Factor for Acute Rejection Occurring during the DGF Period

Rejection episodes that occurred before 1998 were distributed according to their impact on kidney function [21] and those from 1998 to 2014 according to three Banff grades: borderline, grades Ia and Ib (24%), grade IIa (66%), and grade IIb (10%). After including all risk factors listed in Table 1 in a multivariable logistic regression analysis, seven of them were significant, all the others being excluded from the model at *P* > 0.10 (Table 4). Thus, peak HLA sensitization, prolonged pretransplant dialysis period, absence of use of antilymphocyte induction therapy, mycophenolate mofetil and perioperative saline loading, high number of HLA mismatches, and young recipient's age were detrimental for rejection.

### 3.5. Risk Factors for DGF

Continuous variables were categorized to test the linearity of the response to DGF. This response did not depart from linearity for warm and cold ischemia times, recipient's and donor's age, recipient's and donor's BMI, and DCD donors, at variance with recipient's residual diuresis for which 500 mL/d was a significant cut-off value (*P* < 0.001) justifying the creation of a dichotomous variable: <500 (64.5% of grafts) versus ≥ 500 mL/d (35.5% of grafts). The final model included seven risk factors, all the others being excluded at *P* > 0.10: cold ischaemia time, recipient's residual diuresis <500 mL/d, perioperative saline loading, recipient's BMI, DCD donor, donor's terminal creatinine, and recipient's gender. These results were nearly identical whether rejection episodes occurring during the DGF period were taken into account (1155 grafts with complete data [65% of the total]: 976 without DGF and 179 with DGF, upper part of Table 5) or not (1069 grafts [68% of the total]: 911 without DGF and 158 with DGF, lower part of Table 5). Of note, cardiovascular antecedents were more frequent in male than in female recipients: 12.6 versus 8.6% (*P* = 0.01). Restricting the analysis to the 1998–2014 period in which transplants with incomplete data were less frequent (31% out of 978 grafts: Table 1) did not substantially change the significance nor the relative power of the risk factors except for perioperative saline loading, which was not included in this analysis as all recipients benefited from it during that period (results not shown).

**Figure 1:**
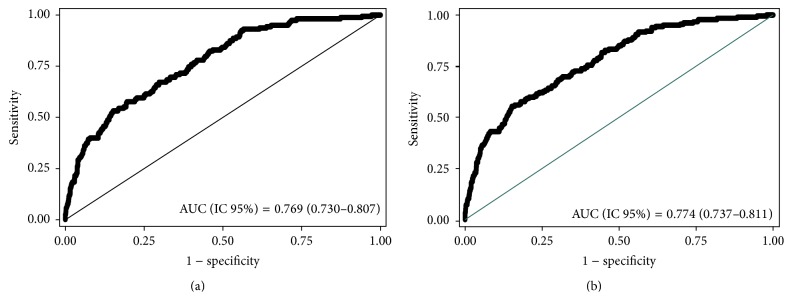
ROC curve derived from the logistic regression analysis of delayed graft function (DGF) with (a) (1155 grafts) and without (b) (1069 grafts) acute rejection episodes (ARE).

ROC curves estimating the discriminant power of the factors which emerged from the logistic analysis were computed. The area under the curve was nearly identical whether rejection episodes were taken into account (0.774, 95% CI: 0.74–0.81, Figure 1(a)) or not (0.769, 95% CI: 0.73–0.81, Figure 1(b)).

### 3.6. Impact of DGF on Patient and Graft Outcome

**Figure 2:**
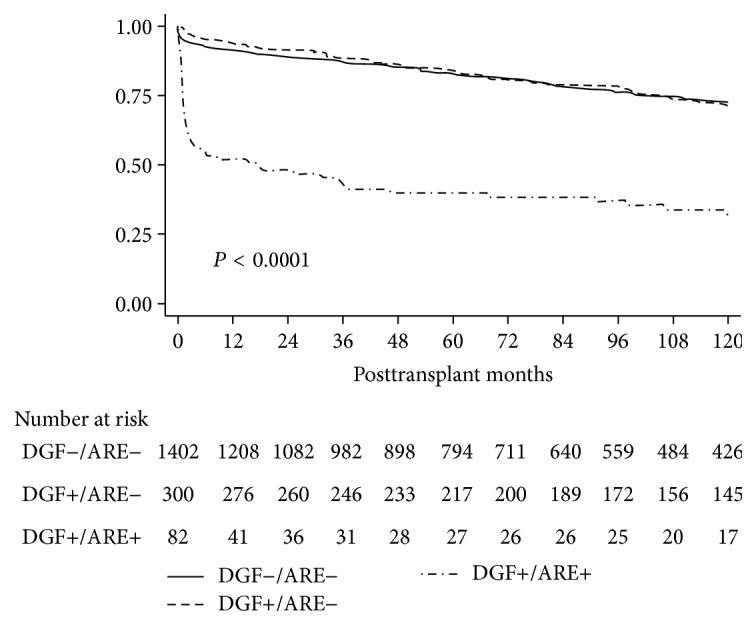
Deceased censored graft survival according to presence (+) or absence (−) of delayed graft function (DGF) and acute rejection episodes (ARE) in 1784 grafts performed from 1983 to 2014.

Survival was studied in three groups according to presence (+) or absence (−) of DGF and ARE: group 1 DGF−/ARE− (*n* = 1402), group 2 DGF+/ARE− (*n* = 300), and group 3 DGF+/ARE+ (*n* = 82). Patient survival was similar in the three groups: at ten years it was 84.5% in group 1, 86.8% in group 2, and 86.5% in group 3 (*P* = 0.54). On the contrary, whereas no difference in deceased censored graft survival was observed between groups 1 and 2 (*P* = 0.45), survival was much lower (*P* < 0.001) in group 3 than in the other two groups (Figure 2), most of this difference being reached during the first year. Multivariate Cox analysis including all risk factors for deceased censored graft survival (see Patients and Methods) yielded similar results (not shown).

## 4. Discussion

Over the past three decades, DGF incidence has consistently fallen due to a better control of its predominant causal mechanisms, ischemia-reperfusion injury, and early occurring rejection episodes. Since the early nineties, these episodes decreased by the use of potent immunosuppressive regimens including mycophenolate mofetil [24] and monoclonal antibodies directed to T cell receptors [25] as well as by a drastic reduction in recipient's HLA sensitization which was closely related to a drop in pretransplant blood transfusions. On the other hand, improved kidney allocation strategy within the US [6] and the Eurotransplant Organization at the end of the nineties [26] has considerably shortened cold ischemia time which constitutes a prominent risk factor for ischemia-reperfusion injury in spite of an increasing but limited rate in detrimental factors such as extended criteria and DCD donors, recipient's diabetes, high BMI, and cardiovascular antecedents [27].

In our centre, implementation of systematic perioperative saline loading since 1992 induced an immediate and impressive drop in DGF incidence that decreased more slowly thereafter, following a trend similar (but at lower values) to that reported in other centres. This observation prompted us to find an explanation for this discrepancy.

In order to distinguish the respective role of ischemia-reperfusion injury and acute rejection occurring during the DGF period, risk factors for rejection during the DGF period were studied. Along with well known factors (HLA sensitization, duration of regular pretransplant dialyses, use of mycophenolate mofetil and antilymphocyte globulin induction, HLA-A+B+DR match, and recipient's age), perioperative saline loading significantly prevents acute rejection. As perioperative saline loading has been shown to decrease the risk of ischemia-reperfusion injury [28, 29] that enhances the susceptibility to rejection by activation of the innate immunity, which is deeply involved in alloimmunity [9], it is likely that perioperative saline loading reduces the immunologic mechanisms that elicit the rejection process.

Risk factors for DGF are very similar whether early occurring rejection episodes are taken into account or not. All these factors except perioperative saline loading are different from those for acute rejection. This suggests that ischemia-reperfusion injury is the predominant mechanism involved in the development of DGF. Several factors contribute to increase in ischemia-reperfusion injury and thereby DGF risk. Whether their effects are additive or enhance each other is difficult to establish. Anyhow, prolonged cold ischemia time, absence of perioperative saline loading, high recipient's BMI, male gender (which is highly correlated to his cardiovascular antecedents), DCD donors, and high donor's terminal creatinine (which is highly correlated to donor's traumatic death) are all detrimental for DGF, consistent with previously reported results [3]. A not yet described risk factor is recipient's residual diuresis. It reflects the function of the native kidneys and is likely to be associated with the nutritional, cardiovascular, and biochemical status of the recipient [30]. Therefore, it is not surprising that posttransplant dialysis is less indicated if recipient's diuresis is preserved.

When early occurring rejection episodes are taken into account, deceased censored graft survival is lower in grafts with DGF than in those without DGF, at variance with what is observed in rejection-free grafts. Long-term graft survival depends mostly on immunologic processes inducing acute rejection [31] and, later on, chronic rejection [32], whereas ischemia-reperfusion injury is short-lived, limited to the first weeks after transplantation. The ambiguity concerning the role of DGF in graft survival is thus cleared away. Poor graft survival attributed to DGF in other studies might be due to unrecognized rejection [10–12, 33] and/or poorly characterized lower intrinsic kidney quality [34]. The differential effects of DGF on graft survival according to acute rejection episodes as well as the longer duration of DGF when it is associated with rejection further suggest that ischemia-reperfusion injury is the predominant factor responsible for DGF development and also that early occurring rejection episodes are to be considered in any study on DGF.

Area under the ROC curves derived from our logistic regression analyses, close to 0.77, shows an excellent discriminant power of the risk factors identified in our model and compares favourably with that reported (0.70) in a large multicentric study [3]. Moreover, restricting the analysis to the 236 grafts performed in our centre between 2003 and 2006, the DGF probability is 11% when using the twenty-two risk factors provided by the transplant calculator derived from the data of 2003–2006 [3] and 7.8% when using our model including seven risk factors. This further highlights the importance of the two novel risk factors which emerged from our study: recipient's residual function of native kidneys and perioperative hemodynamics.

The usefulness of our model should be confirmed by internal validation in new patients using the same database as well as by external validation in patients from other databases using our model [35]. Indeed, our single centre retrospective study has some limitations: (a) the sample size is relatively small when compared with large multicentric studies, (b) DGF incidence is highly dependent on each centre as standardization of postoperative dialysis requirements is lacking, and (c) although our results were similar when restricting the analysis to the 1998–2014 period, it may be argued that recent changes in kidney transplant characteristics render obsolete data derived from a study starting in the eighties.

## 5. Conclusions

The current low DGF incidence in our centre, close to 5%, is, at least in part, related to systematic recipient's perioperative saline loading that efficiently prevents ischemia-reperfusion injury, the predominant cause of DGF. Along with other factors, residual function of the native kidneys is another novel important factor emerging from our study. Our model affords excellent discriminant power to predict DGF. DGF significantly reduces graft survival only when early occurring rejection episodes are taken into account, suggesting that acute rejection should be considered in any study on DGF. As they are powerful determinants of rejection, recent immunosuppressive regimens and better donor-recipient HLA match might further contribute to reduction in DGF incidence.

## Figures and Tables

**Table 1:** Time evolution of demographic characteristics in 1784 transplantations performed from 1983 to 2014.

	Transplant years	1983–1991	1992–1997	1998–2003	2004–2009	2010–2014	*P* value	Number of missing values	
	Number of grafts	421	385	346	353	279		<1998	≥1998

Recipient	Male gender	60.8	61.8	61.9	62.9	62.0	0.99		
	Age (y)	35.8 ± 9.7	39.1 ± 11.1	46.7 ± 12.4	50.6 ± 12.4	53.1 ± 12.8	<0.001		
	Regrafting	20.2	17.1	26.3	13.6	15.1	<0.001		
	Duration of regular dialysis (y)	3.5 ± 3.0	3.7 ± 3.0	4.1 ± 4.6	3.1 ± 2.9	3.5 ± 3.1	0.007		
	History of diabetes	1.1	0	4.0	11.9	15.1	<0.001		
	History of cardiovascular disease	4.8	6.5	10.4	22.4	13.6	<0.001		
	Peak (>50% PRA) sensitization	20.7	6.8	9.2	8.2	6.1	<0.001		
	Delayed graft function (DGF)	48.7	17.7	16.5	11.0	4.7	<0.001		
	Perioperative saline loading	0	100	100	100	100	<0.001		
	Use of mycophenolate mofetil	0	4.9	83.5	92.1	98.9	<0.001		
	Immunoglobulin induction	56.5	95.3	78.6	92.9	98.9	<0.001		
	ARE occurring during DGF period	27.3	10.7	5.8	6.3	3.6	<0.001		
	Biopsy proven	79.8	90.4	94.2	94.6	96.8	<0.001		
	Residual diuresis (<500 mL/24 h)	69.0	66.5	64.5	51.3	52.3	<0.001	267	127
	BMI (kg/m^2^)	22.7 ± 3.8	23.9 ± 3.6	24.5 ± 4.3	25.2 ± 4.7	25.3 ± 4.8	<0.001	271	126

Donor	Male gender	60.9	62.2	59.5	58.4	50.5	0.024		
	Age (y)	52.4 ± 14.3	37.5 ± 14.4	42.7 ± 16.0	44.0 ± 15.1	48.8 ± 13.0	<0.001		
	Donation after cardiac death	0	0	1.2	2.7	13.6	<0.001		
	Traumatic cause of death	57.9	45.7	40.8	34.9	27.2	<0.001	11	8
	Terminal serum creatinine (mg/dL)	1.00 ± 0.39	0.95 ± 0.36	0.91 ± 0.33	0.77 ± 0.31	0.70 ± 0.25	<0.001	43	51
	BMI (kg/m^2^)	23.2 ± 4.0	23.7 ± 3.5	24.7 ± 4.2	24.9 ± 4.6	25.6 ± 4.3	<0.001	132	131

Transplant	Ischaemia times								
	Cold (h)	27.3 ± 7.6	22.8 ± 6.2	18.8 ± 6.0	17.2 ± 5.0	16.3 ± 5.5	<0.001	0	2
	Warm (min)	34.2 ± 7.9	33.0 ± 7.8	33.2 ± 8.3	30.0 ± 7.5	32.6 ± 9.0	<0.001	0	2
	Number of HLA-A+B+DR mismatches	2.6 ± 1.5	2.2 ± 1.3	2.7 ± 1.3	2.5 ± 1.2	2.6 ± 1.1	<0.001		

Nominal variables are expressed as percent of total grafts and numerical variables as mean ± standard deviation.

PRA: panel reactive antibodies;  ARE: acute rejection episode;  BMI: body mass index.

**Table 2:** Delayed graft function (DGF) in transplants with and without acute rejection episodes (ARE) occurring during the DGF period.

Posttransplant weeks	Total DGF		DGF without ARE		DGF with ARE	
	*n*	%	*n*	%	*n*	%
1st	162	42.4	150	50.0	12	14.6
2nd	149	39.0	114	38.0	35	42.7
3rd	39	10.2	23	7.7	16	19.5
4th	12	3.1	6	2.0	6	7.3
>4th	20	5.3	7	2.3	13	15.9

Total DGF	382	100.0	300	100.0	82	100.0

Total transplants	1784		1576		208	

**Table 3:** Relationship between donor's cause of death and terminal serum creatinine.

Cause of death	*n*	%	Serum creatinine mg/dL: m ± SEM
Number of missing data	100	5.6	

Traumatic	691	41.0	0.95 ± 0.01
Cerebrovascular event	837	49.8	0.82 ± 0.01
Anoxia	115	6.8	0.79 ± 0.04
Brain tumor	24	1.4	0.84 ± 0.06
Other	17	1.0	0.85 ± 0.13

Nontraumatic	993	59.0	0.82 ± 0.01

All known causes	1684	100.0	

**Table 4:** Multivariable logistic regression analysis of factors predicting acute rejection episodes occurring during the delayed graft function period in 1784 transplants performed from 1983 to 2014.

Predictive factors	Risk code	Regression coefficient	*P* value	Odds ratio (95% CI)
Constant		−2.119	<0.001	
Peak HLA sensitization (>50% PRA)	If yes, then 1, else 0	1.265	<0.001	3.5 (2.4–5.2)
Duration of regular dialyses (years)	Increase per year	0.098	<0.001	1.103 (1.062–1.146)
Antilymphocyte globulin induction	If no, then 1, else 0	0.860	<0.001	2.4 (1.6–3.4)
Recipient's age (years)	Decrease per year	−0.025	<0.001	0.975 (0.961–0.989)
HLA-A+B+DR mismatches (MM)	Increase per MM	0.206	<0.001	1.22 (1.09–1.39)
Recipient's perioperative saline loading	If no, then 1, else 0	0.624	0.005	1.9 (1.2–2.9)
Use of mycophenolate mofetil	If no, then 1, else 0	0.630	0.007	1.9 (1.2–2.9)

Log likelihood chi-square (7 DF): 238.18, *P* < 0.0001. Goodness of fit: *P* = 0.85.

**(a):** 

Predictive factors	Risk code	Regression coefficient	*P* value	Odds ratio (95% CI)
Grafts with ARE (*n* = 1155)				
Constant		−7.082	<0.001	
Cold ischaemia time	Increase per h	0.091	<0.001	1.094 (1.064–1.124)
Recipient's residual diuresis	If <500 mL/d, then 1, else 0	0.851	<0.001	2.3 (1.6–3.5)
Perioperative saline loading	If no, then 1, else 0	1.200	<0.001	3.3 (2.0–5.4)
Donation after cardiac death	If yes, then 1, else 0	1.292	0.001	3.6 (1.7–7.8)
Recipient's BMI	Increase per kg/m^2^	0.066	0.004	1.068 (1.026–1.113)
Donor's terminal creatinine	Increase per mg/dL	0.730	0.008	2.1 (1.3–3.4)
Recipient's gender	If male, then 1, else 0	0.594	0.009	1.8 (1.2–2.7)

Log likelihood chi-square (7 DF): 163.71, *P* < 0.0001. Goodness of fit: *P* = 0.80.

**(b):** 

Predictive factors	Risk code	Regression coefficient	*P* value	Odds ratio (95% CI)
Grafts without ARE (*n* = 1069)				
Constant		−6.938	<0.001	
Cold ischaemia time	Increase per h	0.092	<0.001	1.096 (1.066–1.128)
Perioperative saline loading	If no, then 1, else 0	1.182	<0.001	3.3 (1.9–5.6)
Recipient's residual diuresis	If <500 mL/d, then 1, else 0	0.843	<0.001	2.3 (1.6–3.5)
Recipient's BMI	Increase per kg/m^2^	0.064	0.003	1.066 (1.022–1.112)
Donor's terminal creatinine	Increase per mg/dL	0.681	0.009	2.0 (1.2–3.3)
Donation after cardiac death	If yes, then 1, else 0	1.079	0.010	2.9 (1.3–6.7)
Recipient's gender	If male, then 1, else 0	0.515	0.012	1.7 (1.1–2.5)

Log likelihood chi-square (7 DF): 137.34, *P* < 0.0001. Goodness of fit: *P* = 0.71.
